# Shelters and Lodging Houses

**Published:** 1904-11-12

**Authors:** 


### The Hospital.

A Journal of

The Medical Sciences and Hospital Administration.

Vol. XXXVII.—No. 946. NOVEMBER 12 1904.

### Shelters and Lodging Houses.

The correspondence, or, as it may perhaps be called, the controversy, now proceeding in the *Times* paper on the general subject of shelters and lodging houses, is one of considerable importance alike to public morals and to public health. The discussion is essentially a very old one; but its revival on the present occasion was due to Mr. John Burns, M.P., who, at a meeting of the London County Council on October 25, strongly opposed a recommendation of the Housing of the Working Classes Committee to establish, at a cost of £50,000, a new lodging house in the vicinity of Drury Lane. Mr. Burns contended that the aggregation of large numbers of celibate vagrants in one spot was not desirable, and declared himself to be "strongly against big lodging houses." He thought the Council had already gone too far in that direction, and their Carrington House in Deptford was not paying. His opposition was ineffectual, and the new house is to be built and opened, but the debate had the incidental effect of calling forth a letter from Mr. Loch to the *Times*, in which he pointed out that the existing shelters for vagrants had not effected any reduction in the amount of vagrancy, vice, and crime, and, on the other hand, that a definite increase in all these evils was at least in part a proof of the inefficient or even injurious character of the means hitherto employed in dealing with them. He declared that the public pays for the existing shelters twice over, and more than twice over, first by the voluntary contributions out of which they are built, and then out of the rates for the poor relief given to many of their inmates. From this condemnation he did not even exempt the Rowton houses, although they differ from the ordinary shelters in many advantageous ways. He quoted Mr. Lockwood, one of his Majesty's Poor Law Inspectors, as his authority for the statement that in the three years ending in 1902 the Rowton house at Hammersmith had cost the guardians £2,900, presumably in relief given to applicants, who were attracted into the parish by its agency; and, while admitting that in some cases much good may be done by the agency of shelters, he nevertheless maintained that it is quite unnecessary to cast a pauperising net far and wide in order to catch men and improve them. "No colony or farm," he says, "justifies shelters or lodging-homes where a large, unclean, unwashed, disease-[illegible]minating population flows in and out, nothing bettered, but rather at each reception grown worse, sleeping in a stifling atmosphere to go out the next morning to a day of idleness in which a few pence, a job, or a chance gift will enable them to purchase yet other days of idleness and nights of sleep in foul and depressing air."

It is only natural that the representative of the Salvation Army, the methods of which were thus criticised, should enter the lists in their defence, and Mr. Bramwell Booth does this from the vantage ground of a general recognition of the earnest desire of the rulers of the Army to deal with the problems of vagrancy and idleness by methods of cure rather than by methods of merely temporary alleviation. It is unquestionable that some of the unfortunate persons who find refuge in the shelters of the Army are enabled by its assistance to work their way back into respectable positions; but this, which nobody disputes, does not really touch the merits of the question. These merits must turn upon the aggregate effect of the shelter, not upon what it may accomplish in particular instances. If the aggregate effect is that a mass of the most degraded examples of humanity are collected together, night after night, in the same locality, already densely populated, is it not inevitable that the very aggregation must intensify the mischief incidental to the presence of even scattered examples of the class? Much has been made of "celibate" tramps, and even these, as the last metropolitan epidemic of small-pox gave only too much proof, are widely "disease-disseminating," but Mr. Bramwell Booth has given, as one of his examples of a rescue effected, the case of a "young workman with his wife and child," and everyone acquainted with our great main roads is aware that "celibacy" is not the ordinary condition of vagrant life. A man and woman usually drag two or more wretched children after them, and there can be no doubt that these children are active agents in the diffusion of the infantile infectious maladies which are accountable, every year, for the deaths of so many thousands of children under five years of age. The question is urgently one of public health as well as of morals and economy, and there can be very little doubt that vagrancy should be restrained, and sternly restrained, by law. If we are not mistaken, the law exists, and needs only to be enforced, or, perhaps, in some respects to be amended. Mr. Bramwell Booth would welcome a power to commit the incorrigibly idle to penal working colonies, and any course of this description would manifestly hinder them from flocking to the doors of shelters in search of supper and a bed. The co-operation between the local authorities of different districts, which may ultimately be expected as an outcome of the meeting convened by Mr. Long, would afford immense facilities for dealing with confirmed vagrancy and for sifting out the men and women who by wisely directed charity might again be raised to better things.

